# Leptin and Aging

**DOI:** 10.18632/aging.100637

**Published:** 2014-02-13

**Authors:** Beatrice M. Filippi, Tony K.T. Lam

**Affiliations:** ^1^ Toronto General Research Institute, University Health Network, Toronto, Canada, M5G 1L7; ^2^ Department of Medicine, University of Toronto, Toronto, Canada, M5S 1A8; ^3^ Department of Physiology, University of Toronto, Toronto, Canada, M5S 1A8; ^4^ Banting and Best Diabetes Centre, University of Toronto Toronto, Canada, M5G 2C4

Aging-associated cellular processes such as oxidative stress and inflammation are accelerated in obesity. An increased in cellular inflammation and oxidative stress also leads to insulin and leptin resistance and a dysregulation of energy homeostasis in obesity, independent of aging [1]. However, aging has been implicated to alter leptin and insulin sensitivity in rodents [2]. These studies underscore the importance in understanding the role of leptin and insulin signaling in aging, obesity, and diabetes.

In this regard, recent literature highlights the integrative role of hypothalamus in leptin signaling, obesity, aging, and diabetes. For example, overnutrition elevates hypothalamic ER stress and activates inflammatory IKKbeta/NF-kappa B pathway, leading to leptin resistance and obesity in rodents [1]. The same hypothalamic inflammatory pathway is activated during aging while direct blockade of hypothalamic inflammation slows down aging in rodents [3]. Whether an enhancement of hypothalamic leptin signaling is responsible for such alteration in longevity remains unknown. However, direct enhancement of hypothalamic leptin signaling in insulin-deficient rodents has been documented to decrease blood glucose levels and increase lifespan [4]. These findings collectively suggest that studies aim to dissect leptin signaling in the hypothalamus may unveil novel therapeutic targets to combat aging-associated metabolic disorders.

Interestingly, metabolic control ignited by leptin is not limited to hypothalamic leptin signaling. Leptin is not only synthesized in the adipocytes but also in the stomach [5] and that gastric leptin is secreted into the intestinal lumen during feeding [5]. Based on these findings, our laboratory recently tested a hypothesis that intestinal leptin signaling regulates glucose homeostasis through a neuronal network [6]. We demonstrated that direct administration of leptin into the small intestine (jejunum section) activates a jejunal long-form leptin receptor-PI3K mediated neuronal network to lower glucose production in healthy, high-fat fed or insulin-deficient uncontrolled diabetic rodents [6]. These findings unveil jejunum as a novel glucoregulatory site of leptin and specifically indicate that enhancing leptin signaling in the jejunum lowers glucose production and blood glucose levels in insulin-deficient diabetic rodents. In analogues to what is described earlier for the metabolic benefits of leptin action in the brain, we postulate that targeting jejunal leptin signaling cascade may delay aging-associated metabolic disorders and increase longevity in diabetes and obesity.

On another note, mid-life obesity correlates with the incident of late-life Alzheimer's disease (AD), which is characterized by increased amyloid plaques and tau phosporylation in the brain. Diet-induced obesity has been documented to accumulate cerebral amyloid plaques while weight loss correlates with decreased cerebral amyloid pathology. Further, low-grade inflammation leads to increased tau phosphorylation and β-amyloid plaques in the brain and neurodegeneration, as well as hypothalamic leptin resistance and obesity as discussed above. Given that leptin decreases tau hyper-phosphorylation in neuronal cells and reduces amyloid-β deposition in the brain, a possibility remains that obesity induces brain inflammation and leptin resistance, leading to an accumulation of amyloid plaques and AD. In fact, a recent clinical study reports that despite an increase of leptin levels in CSF and hippocampal tissues of AD patients, the expression level of leptin receptor in the brain is decreased while leptin receptor protein is localized to neurofibrillary tangles [7]. These data suggest that leptin resistance in the brain correlates with AD. In light of the absolute uncertain role of intestinal leptin signaling in AD, future investigations are warranted to examine the consequence of altering brain and/or intestinal leptin signaling in AD.

Lastly, in addition to the ability of jejunal leptin signaling to sufficiently lower blood glucose levels in diabetes, we have also demonstrated that jejunal leptin signaling is necessary for the anti-diabetic effect of bariatric surgery [6]. Interestingly, a recent parallel study reports that bariatric surgery improves glucose metabolism and reduces the expression of AD markers in the blood mononuclear cells of humans [8]. Given the potential link between leptin and AD as discussed above, it would be interesting to explore whether brain and/or intestinal leptin signaling plays a role in bariatric surgery, obesity, and AD.

In summary, we propose that alteration of leptin signaling in the brain and intestine may provide a mechanistic link between aging, obesity, and diabetes.
